# Cell culture and passaging alters gene expression pattern and proliferation rate in rheumatoid arthritis synovial fibroblasts

**DOI:** 10.1186/ar3010

**Published:** 2010-05-12

**Authors:** Elena Neumann, Birgit Riepl, Anette Knedla, Stephanie Lefèvre, Ingo H Tarner, Joachim Grifka, Jurgen Steinmeyer, Jurgen Schölmerich, Steffen Gay, Ulf Müller-Ladner

**Affiliations:** 1 Department of Internal Medicine and Rheumatology, University of Gießben, Kerckhoff-Klinik, D-61231 Bad Nauheim, Benekestr. 2-8, Germany; 2 Department of Internal Medicine I, University of Regensburg, D-93042 Regensburg, Franz-Joseph-Strauß-Allee 11, Germany; 3 Department of Orthopedics, University of Regensburg, D-93042 Regensburg, Franz-Joseph-Strauß-Allee 11, Germany; 4 Department of Orthopedics, Laboratory of Experimental Orthopedics, University Hospital of Giessen and Marburg, D-35392 Giessen, Paul Meimberg Str. 3, Germany; 5 Center for Experimental Rheumatology, Department of Rheumatology, USZ, CH-8091 Zürich, Gloriastraßbe 25, Switzerland

## Abstract

**Introduction:**

Rheumatoid arthritis synovial fibroblasts (RASF) are key players in synovial pathophysiology and are therefore examined extensively in various experimental approaches. We evaluated, whether passaging during culture and freezing has effects on gene expression and cell proliferation.

**Methods:**

RASF were passaged for up to 8 passages. RNA was isolated after each passage and cDNA arrays were performed to evaluate the RNA expression pattern during passaging. In addition, doubling time of the cells was also measured.

**Results:**

From passages 2-4, mRNA expression did not change significantly. Gene expression in RASF started to change in passages 5-6 with 7-10% differentially expressed genes. After passages 7-8, more than 10% of the genes were differentially expressed. The doubling rate was constant for up to 5 passages and decreased after passages 6-8. After freezing, gene expression of the second passage is comparable to gene expression prior to freezing.

**Conclusions:**

The results of this study show, that experiments, which examine gene expression of RASF and shall reflect or imitate an in vivo situation, should be limited to early culture passages to avoid cell culture effects. It is not necessary to stop culturing SF after a few passages, but to keep the problems of cell culture in mind to avoid false positive results. Especially, when large-scale screening methods on mRNA level are used. Of note, freezing does not affect gene expression substantially.

## Introduction

Predominant features of rheumatoid arthritis (RA) are synovial hyperplasia, synovial cell activation and articular inflammation associated with subsequent cartilage and bone destruction [1]. In this scenario, activated synovial fibroblasts (SF) are key players in joint destruction at the site of invasion into articular cartilage and bone [1-5]. They maintain their aggressive phenotype towards cartilage even when primarily cultured and thereafter co-implanted together with normal human cartilage into immunodeficient severe combined immunodeficient mice (SCID) mice for an extended period of time [5].

To inhibit the progressive growth at the invasion zone followed by cartilage and bone degradation without interfering with physiologic matrix remodeling, identification of pathways operative specifically in RASF and not in SF of other origin (*e.g*. osteoarthritis SF) is essential. Therefore, genes showing a dysregulation that is restricted to RASF are the experimental target of numerous research groups [6-17].

Various strategies, for example differential display, subtractive cell-hybridization, and cDNA arrays and many more, have been developed to examine tissue- and disease-specific differences in gene expression [10,11,18-23]. In addition, a variety of experiments that address the evaluation of pathways of cartilage and bone destruction and their underlying mechanisms were performed with *in vitro *cultured RASF populations isolated from tissue samples obtained during synovial joint replacement. Moreover, to test the effects of new drugs or novel treatment strategies, *in vitro *or in animal models experiments with cultured RASF are essential [8,10,14,24-26].

In contrast to these goals and these experimental approaches, even when the RASF appear activated and 'transformed', they are not fast growing or immortal tumor cell lines, which show a constant geno- and/or phenotype for an extended cultivation period. They are slow to moderately proliferating cell populations, which during cultivation may alter their *in vivo *phenotype when devoid of their normal environment. In addition, in contrast to fast-proliferating tumor cells, in RA only limited amounts of synovial cells, and therefore limited amounts of mRNA, can be obtained for molecular analysis. Therefore, the cells are often grown over several passages to obtain sufficient cellular material to perform the required experiments. In this situation, it is frequently difficult to know whether the cell population after an extended cultivation time is still identical to the RASF population shortly after isolation from the tissue. Moreover, passaging may result in a selection pressure for parts of the cell population, for example adherent cells vs. trypsin-sensitive cells, that are being removed to a different extent from the culture flask during passaging and that may alter the overall gene expression profile in higher passages and lead to different results when compared with earlier passages.

To evaluate whether cell culture effects take place in RASF cultures over several passages, we performed cDNA array analysis for up to nine passages. Early passages were compared with later passages to evaluate alteration of gene expression as consequence of cell culture in detail. In addition, the proliferation rate was measured by the doubling time of the population over the passaging time of the cells to evaluate changes in cell growth rates in comparison to earlier passages.

## Materials and methods

### Synovial tissue and cell culture

Synovial tissues were obtained from synovial biopsies of six patients with RA undergoing joint surgery (synovectomy or joint replacement by prosthesis implantation), who all met the criteria of the American College of Rheumatology [27]. The tissue samples were obtained during routine surgery at the Department of Orthopedics of the University of Regensburg, where approved by the local ethics committee and patients involved gave informed consent. Culture of SF was performed as described recently [5]. Following enzymatic digestion, fibroblasts were grown in DMEM (Biochrom, Berlin, Germany) containing 10% heat inactivated FCS (Gibco Life Technologies, Grand Island, NY, USA), 100 U/ml penicillin and streptomycin (PAA Laboratories GmbH, Linz, Austria) and cultured for four passages at 37°C in 10% carbon dioxide. The SF were stained for a fibroblast marker by immunohistochemistry. More than 95% could be stained positively for the fibroblast enzyme prolyl 4-hydroxylase and none were positive for the macrophage marker CD68 or the neutrophil marker cathepsin G after the second passage of cultivation with enzymatic digestion equalling passage 0 (data not shown). Routine tests for mycoplasms were negative. At 85 to 95% confluency, cells were passaged 1:2 and a part of the cells was harvested. Total RNA was extracted and stored at -70°C. Culture conditions were (and have to be) kept constant during the experiments. Passaging of the cells was performed at 85 to 95% confluency as fibroblasts exhibit contact inhibition (unpublished observations) and were passaged 1:2 to provide cell-cell contacts between the cells.

### Cell culture proliferation measurement

After enzymatic digestion of the tissue (passage 0), the cells were grown to 85 to 95% confluence, then trypsinized, counted and 50,000 cells were seeded into five fresh cell culture six-wells (passage 1). Each day, one well was trypsinized and the cells were counted. The day at which the double cell number (>100,000) occurred was noted. At 85 to 95% confluency, this procedure was repeated (passage 3) until passage 8. The doubling time in days was determined as 'doubling point', when the cells doubled their number (100,000) as compared with the day they were counted (50,000) and seeded.

### Storage of cells in liquid nitrogen

For evaluation of the effects of freezing, that is storage in liquid nitrogen, cells in passage 1 (the passage after enzymatic digestion) were trypsinized, centrifuged and resuspended in FCS with 10% DMSO (dimethyl sulfoxide) (v/v) in a 1 ml cryovial. Cells were immediately stored on ice, placed in a freezer and frozen over night at -80°C (freezing speed about 1°C/min). Thereafter, cells were directly transferred to liquid nitrogen.

To thaw the cells, the cryovials were carefully thawed at 37°C, the cell suspension transferred into preheated (37°C) culture medium and centrifuged to remove the DMSO. It was resuspended in culture medium and then transferred into cell culture flasks and cultured (passage 2 for the cells after cryostorage) and passaged for up to six passages under standard conditions as outlined above.

### RNA extraction

Total cellular RNA was extracted from human fibroblasts using the RNeasy spin column purification kit (Qiagen, Hilden, Germany). To remove contaminating genomic DNA, total RNA was treated with DNase I (0.2 U/μl; Boehringer Mannheim, Mannheim, Germany) for 40 minutes at 37°C. RNA concentrations were measured using the Ribogreen RNA quantification kit (Molecular Probes, Leiden, the Netherlands), adjusted to 200 ng/μl in water and stored at -70°C. Equal aliquots were then electrophoresed on 1% agarose gels stained with ethidium bromide to compare large and small rRNAs qualitatively and to exclude degradation. When starting with fresh RNAs, one RNA arbitrarily primed(RAP)-PCR was performed (details see below) without the reverse transcriptase as a control for DNA contamination.

### RNA arbitrarily primed PCR of total cellular RNA

RAP-PCR of total cellular RNA was performed as described previously [6,11,22]. As a template for each experiment, 250 ng of RNA was used. First-strand synthesis was carried out using MuLV (Moloney murine leukemia virus) reverse transcriptase (Promega, Madison, WI, USA) and 2 μM first strand arbitary primer. Second strand synthesis was performed in a 20 μl reaction using AmpliTaq Stoffel Fragment (Perkin Elmer, Norwalk, CT, USA), 2.8 μl [α-^32^P]dATP (3,000 Ci/mmol, 10 mCi/ml), and 4 μM arbitrary second primer. Subsequently, the reaction was cycled through 30 low stringency cycles (30 seconds 94°C, 30 seconds 35°C, 30 seconds 72°C). Primer combination for RAP-PCR was: OPN23 (5'-CAG GGG CAC C-3') for first strand and OPN21 (5'-ACC AGG GGC A-3') for second strand synthesis.

### Atlas™ cDNA expression array

Two different AtlasTM human cDNA expression array membranes (Clontech, Palo Alto, CA, USA) containing the human cDNAs were used: The Atlas Human Cancer cDNA Expression Array and the Atlas Human Oncogene cDNA Expression Array. From each of these genes, cDNA was amplified using RAP-PCR as described recently [10,11].

#### Preparation of cDNA probes

The PCR products were purified from unincorporated ^32^P-labeled nucleotides and small cDNA fragments (<0.1 kb) by column chromatography using NucleoSpin^® ^Extraction Kit (Clontech, Palo Alto, CA, USA) as outlined by the producer. A total volume of 100 μl was used for hybridization. A 2 μl sample of the purified probe was measured by scintillation counting.

#### Hybridization

The cDNA was hybridized to the Atlas™ human cDNA expression array membranes in roller bottles. The filters were transferred to roller bottles and prehybridized in 5 ml prewarmed (68°C) hybridization solution (ExpressHyb Hybridization Solution, Clontech, Palo Alto, CA, USA) with 100 μg/ml fragmented denatured salmon sperm DNA in a hybridization oven. The labeled cDNA probe was diluted 1:10 with 10 times denaturing solution (1M NaOH, 10 mM EDTA) and incubated at 68°C for 20 minutes. A 5 μl (1 μg/μl) sample of sheared human genomic DNA was added with an equal volume of two times neutralizing solution (1 M NaH_2_PO_4_, pH 7.0) and incubated for 10 minutes at 68°C. The mixture was added to the filters with the hybridization solution and hybridized over night.

### Wash

The filters were washed three times in wash solution 1 (2 × SSC (saline sodium citrate) and 2% SDS) for 30 minutes at 68°C each. Two washing steps were performed with wash solution 2 (0.1 × SSC and 0.5% SDS) at 68°C for 20 minutes and one step for five minutes at room temperature in 2 × SSC and then exposed to a Phosphor-Imager-Screen (Molecular Dynamics, Sunnyvale, CA, USA) for three to five days depending on the intensity of radiation of the bound fragments. Data analysis was performed using the Ambis software (ImageQuant, Molecular Dynamics, Sunnyvale, CA, USA). Evaluation was performed using the AtlasImage™ 2.7 software, developed specifically for analysis of the Atlas™ cDNA Expression Arrays (Clontech, Palo Alto, CA, USA [28]). Data comparison data have been deposited in the NCBI GEO database with the series record access number [GEO:GSE21385].

### Array comparison and statistical evaluation

To compare arrays of different hybridizations using the Atlas™ array system, background and signal intensity need to be normalized. The default signal threshold determined by the software was used and kept constant for all analyses. After background correction of the arrays, the median signals for all spots on an array were used to calculate the correction coefficient (global normalization), which demonstrated to be the most reliable method for the AtlasImage 2.7 software for our array settings [28].

Statistical evaluation for multiple array comparison was performed using Lavene-test followed by *t*-test (parametric) or by Mann-Whitney U test (non-parametric), with significance level correction according to the number of compared genes (Bonferroni adjustment) as described previously [28]. For 100 comparisons, a random significance of *P *< 0.05 in 5 of 100 comparisons occurs (α-Factor). Therefore, the significance level was adjusted: *P *= 0.05/n_comparisons _(e.g for 100 comparisons the new significance level is *P *< 0.0005). Only genes that reached the statistical significance level after Bonferroni-correction were regarded as being differentially expressed.

### Real-time PCR

Real-time PCR was performed using a LightCycler system (Roche Diagnostics, Mannheim, Germany). Reactions were performed in a 20 μl volume with 0.5 μM primers; CD82 for 5'-TAT GTC TTC ATC GGC GTG GG-3'; CD82 rev 5'-CAT GAG CTC AGC GTT GTC TG-3'; c-myc for 5'-CTA TGA CCT CGA CTA CGA CT-3'; c-myc rev 5'-CGC AGA TGA AAC TCT GGT TC; 18S-for 5'-TCA AGA ACG AAA GTC GGA G-3'; 18S-rev: 5'-GGA CAT CTA AGG GCA TCA CA-3'), 3 mM MgCl_2 _concentration, and 2 μl LightCycler-FastStart Reaction Mix SYBR Green I (Roche Diagnostics, Mannheim, Germany). After 10 minutes polymerase activation at 95°C, 40 cycles with 95°C for 15 seconds, 52°C for 5 seconds and 72°C for 20 seconds were performed. Fluorescence was measured at the end of the 72°C extension period. Efficiencies of the primers were tested using the standard curve method (E = 10^-1/slope^). According to the guidelines of the manufacturer, efficiencies of 2.00 ± 0.05 were considered acceptable for experiments. To confirm amplification specificity, the PCR products were subjected to a melting curve analysis to exclude primer dimers and non-specific amplification. Data were analyzed using the LightCycler analysis software (Roche, Mannheim, Germany). The baseline of each reaction was equalized by calculating the mean value of the five lowest measured data points for each sample and subtracting these values from each reading point. Background fluorescence was removed by setting a noise band. In this setting, the number of cycles at which the best-fit line through the log-linear portion of each amplification curve intersects the noise band is inversely proportional to the log of copy numbers.. The crossing points (CP) are the intersections between the best fit lines of the log-linear region and the noise band. The CP determined for the respective genes were normalized to those of 18S RNA to compensate for variabilities in the amount of RNA and for exclusion of general transcriptional effects.

## Results

### Expression of genes in RASF during cell culture passaging

**Figure 1:**
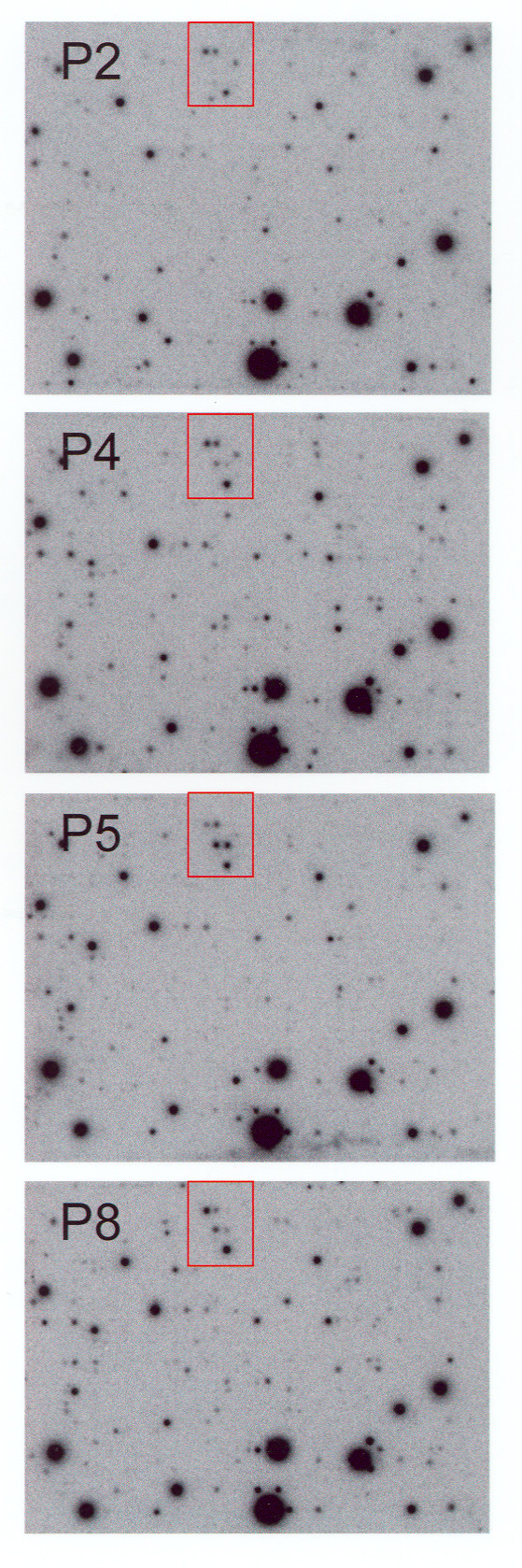
**Expression patterns of genes in RASF during the different cell culture passages**. Example for cDNA arrays of the rheumatoid arthritis synovial fibroblasts (RASF) in different passages from one RA patient. Sections of the Atlas Human Cancer cDNA Expression Arrays are shown. **(a) **Passage 2, **(b) **passage 4 with mostly constant expression when compared with passage 2, **(c) **passage 5 with changes about 7% of the expressed genes when compared with passage 2, and **(d) **passage 8 with changes about 10% of the expressed genes when compared with passage 2. p, passage.

**Figure 2:**
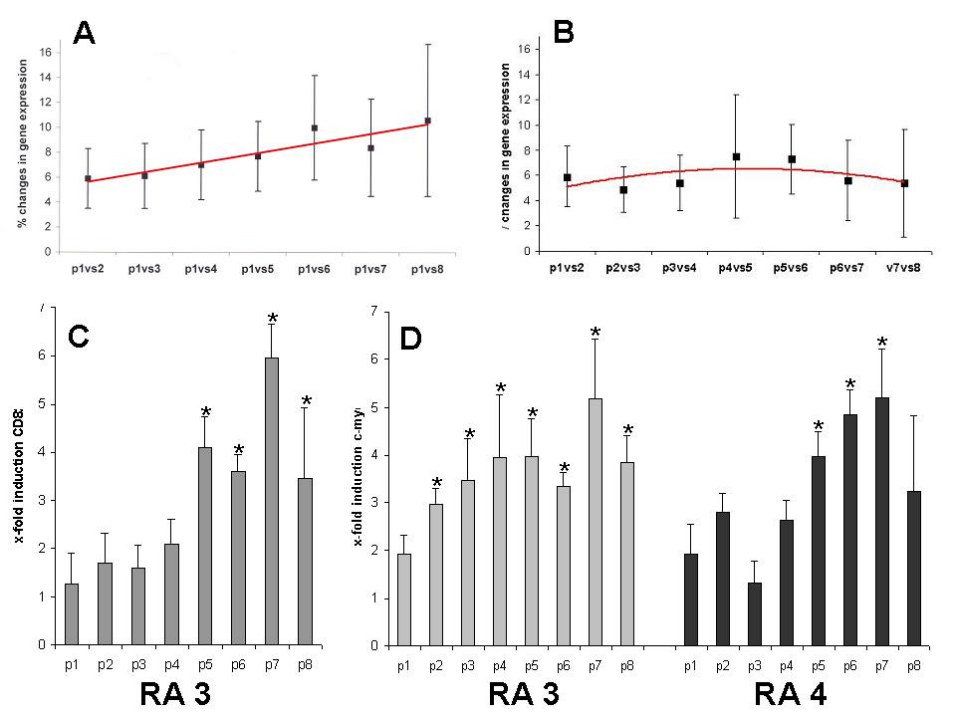
**Changes in gene expression during passaging**. **(a) **The changes in gene expression increase substantially during passaging of the cells in all fibroblast cultures. In addition, the variations and differences between expression pattern in the different rheumatoid arthritis synovial fibroblasts (RASF) cultures increase also in later passages (% changes of gene expression ± standard error of the mean). The regression line shows a clear increment in the course of the culture of the cells (red). **(b) **Changes in gene expression comparing higher passages instead of passage 1. **(c) **Verification of CD82 and c-myc regulation using real-time PCR. Passaging was performed in two parallel cultures for each fibroblast population. Real-time PCR was performed threefold (six measurements for each population, respectively).

For each RA patient, the isolated SF cell population was passaged in two independent experiments. RNA was extracted and cDNA array experiments were performed. The cDNA arrays were subsequently hybridized with radioactive labeled cDNA probes from the different passages of the RA patients (Figure 1). Expression patterns of RASF in early passages in comparison to higher passages was performed for each patient individually (n = 4) and the alteration in percentage was calculated and compared for the four patients (Figure 2). Only genes, that reached the signal threshold, were constant in the repeated experiments, and reached the statistical significance level after Bonferroni-correction as described were regarded as differentially expressed.

The gene expression patterns of the RASF populations were constant in all patients during passages 1 to 4 (about 7% difference in gene expression; Figure 2), and one patient showed even a constant gene expression for up to five passages (data not shown).

Of note, passage 0, which is the first culture of adherent cells after enzymatic digestion (after removing of the non-adherent cells and the supernatant and after several washing steps) differed in the expression pattern to each of the passages 1 to 9 (>10%), indicating that contaminating cells such as macrophages and endothelial cells are, as expected from immunocytochemistry, present at passage 0 (data not shown). In detail, the variation of two parallel cultures of the same RASF populations showed a differential gene expression of below 1% of the expressed genes.

Substantial changes of gene expression in the RASF populations could be detected after passages 5 to 6. They varied individually for the different patients resulting in alteration of about 7 to 10% of the analyzed genes (Figure 2a). After passages 7 to 8, more than 10% of the analyzed genes could be found to be differentially expressed (Figure 2a). The increase in expression changes, altered during passaging, are constant through the passages (Figure 2b). When comparing higher passages with passage 2 instead of passage 1, the same increase of expression changes in percentage could be detected (regressionline in Figures 2a and 2b).

**Figure 3:**
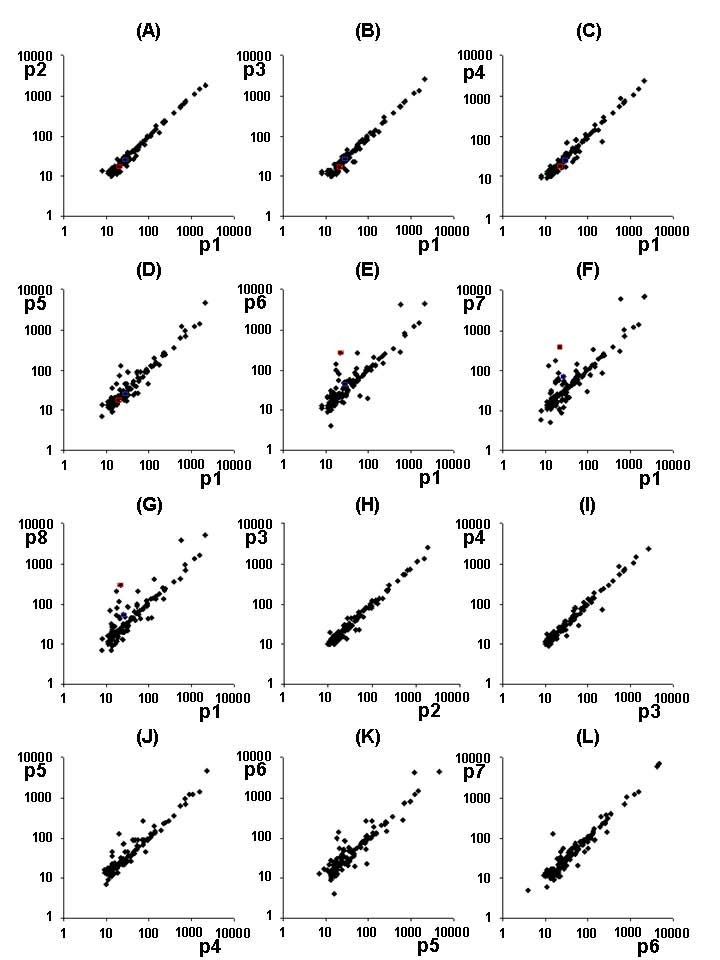
**Changes in gene expression**. Results are presented for one rheumatoid arthritis (RA) patient using an Atlas Human Cancer cDNA Expression Array for direct comparison of the relative intensities of the compared passages, showing strong variations at higher passages in this example. **(a to g) **Passage 1 was compared with higher passages. Examples of two genes are presented. In red: Expression of c-myc. In blue: expression of p33ING1 during culture of the exemplary RA synovial fibroblasts (SF) population. **(h to l) **In addition, higher passages are compared with each other showing the differential expression pattern between higher passages.

Interestingly, the genes that were differentially expressed at later passages were not identical in the different passages, thus underlining the observation that the gene expression pattern starts to be inconstant and diverging in later passages (data not shown). To visualize the changes between the passages, intensities of the arrays compared (after background correction) are presented for one exemplary passage comparison (Figure 3). In addition to the comparisons between early passages (passage 1) and higher passages, the graphs for comparisons between higher passages are also presented. Of note, the differences between the higher passages (e.g. passage 6 to 7) show a more constant pattern then when compared with very early passages (e.g. passage 1 to 7). Altered genes included, for example, oncogenes, cytokines, and proliferation-associated genes, but no specific gene groups were differentially expressed in all patients and all experiments. Examples of the regulated genes and gene groups are listed in Table 1. The list of regulated genes is available at the *Arthritis Research and Therapy *homepage. Here, the genes are presented, in which the differential expression starting in a defined passage reaches statistical significance and differential expression continues through passaging of the cell population (>'*passage'*). In addition, two genes were verified using real-time PCR (c-myc oncogene, CD82), indicated with ** for the RA SF populations tested in Figure 2c. Only genes that reached the signal threshold, were constant in the repeated experiments, and reached the statistical significance level after Bonferroni-correction as described were stated as differentially expressed. Some of the presented genes are highlighted in Figure 3, to illustrate the constancy between the passages in one exemplary fibroblast population. The data reflect, in part, the slowed cell cycling and reduced proliferation, as most of the regulated genes could be related to this process. But also other genes, such as cytokine receptors and regulators of other cellular pathways are altered during passaging (Table 1).

**Table 1:** Genes, that are differentially expressed during cell culture

Gene group	Regulated genes	RA-1		RA-2		RA-3		RA-4	
Cytokines/-receptors	TNF receptor 1	3	↓	5-8	↑	5	↑	3-8	↑
	MCSF I receptor precursor	6,8	↑	6-8	↓	2	↓	5-8	↓
	epidermal growth factor receptor 1	3,4	↑	-		6-8	↑	4-8	↑
	insulin-like growth factor I receptor	>5	↑	3,7	↑	7-8	↑	5,6,8	↑
Adhesion	ILK	>5	↓	2	↑	2,6	↑↓	4-8	↓
Apoptosis	Fas-activated serine/threonine kinase cytotoxic TRAIL receptor 2 (DR 5)	4-8	↑	7-8	↑	6	↑	6-8	↑
		5-8	↑	2	↓	7-8	↓	8	↑
Proliferation	tumor suppressor LUCA1	6-8	↑	7-8	↑	7-8	↑	6,8	↑
	p33ING1	4-8	↑	6-8	↑	3-8	↑	3-8	↑
	p53 cellular tumor antigen	4-8	↑	6,8	↑	3,7	↑	7-8	↑
	cyclin-dependent kinase inhibitor 1C	6-8	↑	2	↑	4-8	↑	-	
	ski oncogene	7-8	↑	4	↑	6,7	↑	6,8	↑
Proliferation/Signaling	shb proto-oncogene	4-8	↓	7-8	↓	-		2	↓
Signaling	notch2	5-8	↑	6	↑	5,6,8	↑	5	↑
	jun-B	7-8	↑	4,5,7,8	↑	5-8	↑	4,7	↑
	frizzled	7-8	↑	8	↑	2	↑	8	↓
	FRA2	3-8	↓	7-8	↓	5-7	↓	2	↑
	NOTCH1 precursor	5-8	↑	5	↑	4,5	↑	7-8	↑
Others	semaphorin 1	7-8	↑	-		4-8	↓	3,4	↑
	glycogen synthase kinase 3 alpha	6-8	↑	6	↑	5-8	↑	3-8	↑
	thymosin beta-10	5,6,8	↓	4,6,7	↓	7-8	↑	5-8	↑
	ezrin; cytovillin 2; VIL2	7-8	↓	4-8	↓	4	↑	5	↓

Exemplary gene groups and genes and their regulations are shown. Only genes, that reached the signal threshold and the statistical significance level after Bonferroni-correction as described in methods were regarded as differentially expressed (*P *< 0.00025).

FRA2: fos-rrelated antigen 2; ILK: integrin-linked kinase; MCSF: macrophage colony-stimulating factor; RA: rheumatoid arthritis; TRAIL: tumor necrosis factor related apoptosis inducing ligand; VIL2: villin 2.

### Expression of genes in RASF after storage in liquid nitrogen

**Figure 4:**
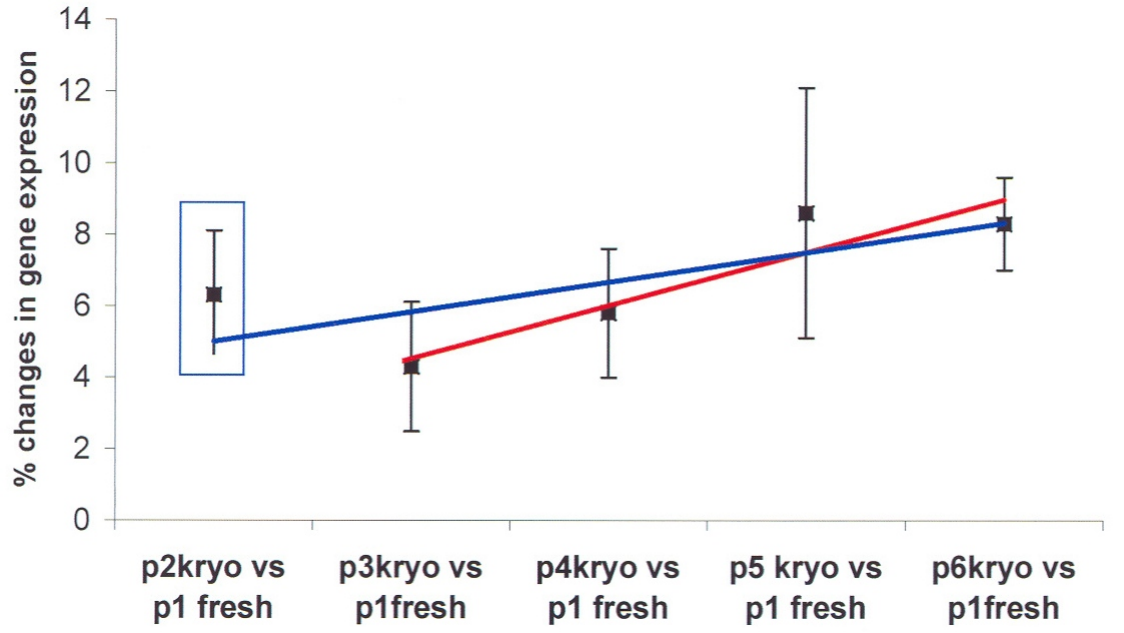
**Changes in gene expression after storage in liquid nitrogen**. The changes in gene expression after thawing the cells and culturing for up to six passages were compared with the freshly cultured cells with passage 1 as baseline for the comparison. The blue regression line shows the values for passage 2 to 6 after thawing of the cells when compared with passage 1 of the freshly cultured cells, including passage 2 (blue square). The red regression line shows the values for the passages 3 to 6 without the first passage after thawing of the cells (excluding the value in the blue square), showing that the passage immediately after thawing showes higher values of differentially expressed genes due to the thawing procedure. p, passage.

The cDNA arrays were subsequently hybridized with radioactive labeled cDNA probes from freshly cultured RASF for up to six passages after thawing of the cells. Expression patterns of the thawed then cultured RASF were compared with the freshly cultured cells (with passage 1 as baseline for constant gene expression basis) as shown in Figure 4. The changes in the gene expression pattern after thawing were similar to the not frozen cells (Figure 2a), and the first passage after thawing (passage 2) showed more changes in gene expression when compared with passage 3 (Figure 4). Substantial changes of gene expression in the RASF populations could be detected in passages 5 to 6 with alterations from 9.5 to 12.0% of the expressed genes (Figure 4).

### Proliferation of RASF during cell culture passaging

**Figure 5:**
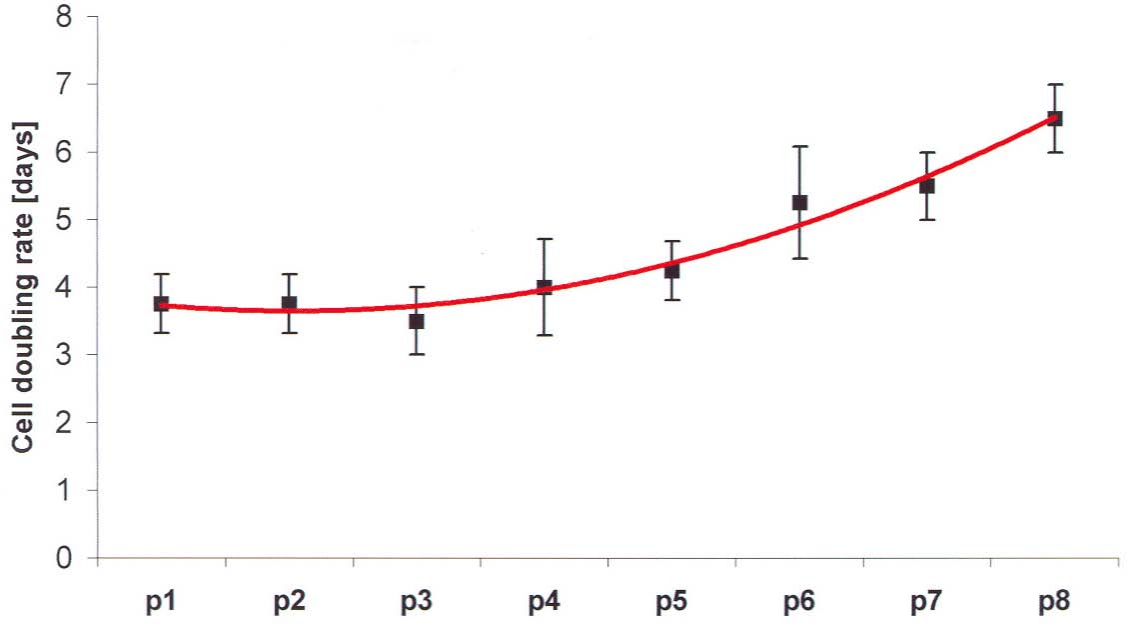
**Cell doubling rates (in days) during cell culture passages for each patient**. The cell doubling rate was measured by counting the cells. The day, the cell numbers were doubled after passaging of the cells was noted for each passage. The tendency shows a constant doubling rate in early passages, which increases during the cultivation of the cells. p, passage.

The cell doubling rate was measured during passages 1 to 8 as described above by counting the cells each day (parallel experiments with equal cell numbers per well as described in methods). The day after the passaging of the cells in which the cell number doubled after passaging was noted for each passage. Interestingly, a constant doubling rate was found in the early passages 3 to 4 and in most patients in passage 5 (4 ± 1 day), which increased during the further cultivation of the cells (passages 6 to 8). At later passages the doubling rate was increased up to seven days, indicating that the 'older' cell populations show a decreased proliferation rate (Table 2 and Figure 5).

**Table 2:** Cell doubling rates (in days) during cell culture passages for each patient

Patient	p1	p2	p3	p4	p5	p6	p7	p8
RA-1	3	4	3	5	5	6	5	7
RA-2	4	3	4	4	4	4	5	6
RA-3	4	4	3	4	4	5	6	7
RA-4	4	4	4	3	4	6	6	6
Mean	3.75	3.75	3.5	4.0	4.25	5.25	5.5	6.5
± SD	0.43	0.43	0.50	0.71	0.43	0.83	0.50	0.50

The cell doubling rate was measured by counting the cells. The day, the cell numbers were doubled after passaging of the cells is listed for each passage.

p: passage; RA: rheumatoid arthritis; SD: standard deviation.

## Discussion

T-cell independent pathways, such as upregulation of proto-oncogenes, production of growth factors and the release of matrix-degrading enzymes lead to progressive destruction of the affected joints [2,15]. Transformed-appearing, activated SF are key players in this synovial activation [1,2,5]. To identify the underlying mechanisms of the destructive behavior of RASF, *in vitro *cultured RASF are used in various experimental settings [10,11,13-15]. The analysis of the pathways that may help to understand the progressive growth at the invasion zone and the active cartilage and bone degradation without interfering with physiologic matrix remodeling is therefore mandatory to identify novel therapeutic targets to inhibit the progressive joint destruction by RASF.

Several cytogenetic and molecular biology techniques are currently used to identify differentially expressed genes under different biological conditions, for example differential display, subtractive hybridization, and cDNA arrays [9,11,13,19,29]. They are currently used to analyze molecular changes and mechanisms involved in the pathogenesis of RA [9-11,30-32]. The advantages of the current techniques include analysis of gene subsets, comparison of more than two biological conditions in combination with a high sensitivity. In additon, to control the effects of potential new drug targets such as proliferation inhibitors, antiinflammatory and destruction inhibiting molecules, *in vitro *analysis using RASF or animal models including the use of human RASF such as the SCID mouse model for RA are helpful tools [9,10,18,25,26,32,33]. Unfortunately, high amounts of cellular material (mRNA or protein) are required in most cases for the different gene or gene product analysis.

To evaluate the critical effects of this passaging procedure in cell culture on gene expression, RASF were therefore cultured over several passages followed by cDNA array analysis for up to eight passages and comparison of early passages to higher culture passages was performed. In addition, the effects of the storage of RASF in liquid nitrogen on gene expression were examined.

As shown in Figure 2, the gene expression pattern of the RASF populations were constant at passages 2 to 4. Passage 0 was different when compared with passages 1 to 8 (>10%), which is most likely due to the presence of macrophages on the culture plate (detectable by immunohistochemistry [1,5]), which are not present at the later passages 1 to 8. In addition, changes in gene expression of RASF populations could be detected after passages 5 to 6, showing changes of 7 to 10% of the analyzed genes (Figure 2). After passages 7 to 8, more than 10% of the analyzed genes were differentially expressed combined with an increasingly inconstant expression pattern at higher passages.

Thawing of the cells after storage in liquid nitrogen affected mainly the gene expression of the first passage of the cells, possibly due to the stress of thawing and the remaining DMSO until the DMSO was completely removed. Thereafter, the cells showed a similar pattern of gene expression when compared with the freshly cultured RASF as the non-frozen cells (Figures 2 and 3). Moreover, the proliferation rates of the fibroblast cultures decreased in later passages, showing a decreased doubling rate after five to eight passages (Table 2).

Taken together, the data of the study show that experiments, which involve analysis of gene expression and the phenotype of RASF, should be limited to early cell culture passages, that is passages 2 to 5, to avoid cell culture effects, diverging gene expression at higher passages, and decreased proliferation of the analyzed RASF populations. In case of a need for larger cell numbers, for example for transduction or animal experiments, an internal long-term cultivation control should be performed, which also includes the comparison of early passaged cells to later passaged cells. In addition, storage of cells in liquid nitrogen affect mainly gene expression of the first culture passage after thawing of the cells and therefore, the second passage should be used for experiments.

Therefore, this paper addresses researchers who perform experimental approaches with cultured SF on the RNA expression level. We want to highlight that culturing of the cells for a too high number of passages will produce differences in gene expression in comparison to the cells used at low passages. Many researchers address the ability to proliferate, the induction of apoptosis and the cytokine expression by these experiments. The intention of the paper is not to recommend excluding culturing RASF after four or five passages, but to keep the problems of culturing in mind to avoid false-positive results and additional, rather labor-consuming, work when verification of the obtained expression data with fresh cell cultures is performed. Culture conditions should be kept constant during the experiments. In addition, we want to emphasize, that the functional ability of the cells or the regulation on protein level are not necessarily changed.

## Conclusions

The increased potential of high-resolution molecular analysis techniques for evaluation of cultured synovial cells, not only reveals the effects of culture on gene expression, it also illustrates the mandatory duty to take these effects into account when simulating the *in vivo *situation with an *in vitro *setting.

## Abbreviations

CP: crossing points; DMEM: Dulbecco's modified eagle medium; FCS: fetal calf serum; RA: rheumatoid arthritis; RAP-PCR: RNA arbitrarily primed PCR; SCID: severe combined immunodeficient mice; SF: synovial fibroblasts.

## Competing interests

The authors declare that they have no competing interests.

## Authors' contributions

EN was involved in the experiment organization, design, and performance; writing, design and structure of the paper. BR performed part of the experiments. AK was involved in the evaluation and interpretation of data. SL was involved in evaluation and interpretation of data. IHT was involved in evaluation and interpretation of data. JG was involved in preparation of the synovial tissue for RASF isolation. JSt was involved in preparation of the synovial tissue for RASF isolation. SG was involved in evaluation and interpretation of data. JSc was involved in evaluation and interpretation of data, structural organziation of the paper. UML was involved in experimental design and organization, paper design and structure. All authors read and approved the final manuscript.
